# Spotlight on Maternal Perceptions of Child Behavior: A Daily Diary Study with Child Welfare-Involved Mothers

**DOI:** 10.3390/bs12020044

**Published:** 2022-02-11

**Authors:** Christina M. Rodriguez, Paul J. Silvia

**Affiliations:** 1 Department of Psychology, University of Alabama at Birmingham, Birmingham, AL 35294, USA; 2 Department of Psychology, University of North Carolina at Greensboro, Greensboro, NC 27412, USA; p_silvia@uncg.edu

**Keywords:** child abuse, parenting, perceived child behavior, experience sampling methods, ecological momentary assessment, daily diary

## Abstract

Research has documented a variety of factors—including stress, attributions, and anger—that may increase parents’ risk for child maltreatment, but most of this research is based on low-risk, community samples of parents’ perceptions about themselves and their children. Moreover, parents are usually asked to provide self-reports wherein they summarize their general impressions distal from actual parenting. The current study employed experience sampling methods with a high-risk sample. Mothers identified for child maltreatment reported on their stress and coping as well as their perceptions regarding children’s misbehavior and good behavior using end-of-day surveys for up to four weeks. Only maternal reports of children’s good behavior based on personality and mood were relatively stable; stress, coping, and reports on child misbehavior varied considerably across days, implying that contributors to daily fluctuations in these factors could represent intervention targets. Although maternal perceptions of misbehavior severity, anger, and negative attributions were interrelated, only anger about misbehavior related to maternal stress levels. Mothers who reported better coping perceived their child’s behavior more favorably that day and were more likely to ascribe positive behavior to the child’s mood and personality. Current findings highlight the importance of positive coping mechanisms in parental perceptions of children; such findings should be replicated to determine how to maximize parental resources that reduce child maltreatment risk.

## 1. Introduction

Child maltreatment is recognized as a critical public health concern worldwide [1]. Child welfare services—the agency tasked with investigating and identifying cases of child maltreatment in the U.S.—received 4.4 million referrals in 2019, with 656,000 children either substantiated or indicated victims of maltreatment [2]. However, given high levels of under-reporting, U.S. prevalence rates estimate 1.25 million children experience maltreatment annually [3], with evidence that substantially more child maltreatment occurs than what is reported through official channels [4]. Furthermore, research using new approaches that track families across time reveal that one of every eight U.S. children (12.5%) will be confirmed as a victim of maltreatment before they reach age 18, which is a cumulative estimate exponentially higher than national annual rates of officially reported child maltreatment cases convey [5]. Therefore, understanding the conditions in which child maltreatment arises remains a critical priority for research and public policy.

Abuse prevention strategies can be designed to prevent recurrence of maltreatment by parents already identified by protective services—a secondary prevention approach—or to avert maltreatment before it ever arises—a primary prevention approach [6]. In order to prevent maltreatment, researchers and practitioners attempt to estimate a parent’s child *abuse risk* based on their beliefs and behaviors that can reliably predict the likelihood of the occurrence or recurrence of child [7,8].

Parents may engage in child maltreatment when they are unable to handle demands, consistent with a stress and coping theoretical framework [9,10]. In this classic conceptualization, stress arises from an appraisal process that then evokes a response (coping) to the stressor [9]. One of the most robust predictors of parental child abuse risk that has been documented worldwide is parents’ stress e.g., [11,12,13,14,15], including differences in stress between parents who have versus have not been identified as abusive [11], and data on parental stress from longitudinal studies [13]. Although protective factors are empirically examined less often, parents’ stronger coping skills have been linked to their lower child abuse risk [16,17], including longitudinal studies tracking coping across time [18], with evidence that better emotion regulation and frustration tolerance reduce the likelihood of child abuse [15,19]. However, how stress versus coping may contribute to perceptions of children’s behavior by parents who are at risk to abuse remains unclear, although such parental perceptions likely influence parent-child conflict.

Current research indicates parents with greater risk to abuse are more likely to perceive problematic behaviors in their children, an effect that has been observed in both community samples of parents [13,14,20,21] as well as clinical samples of at-risk children [22,23]. Longitudinal work suggests that parents reporting elevated stress are more likely to later report greater child behavior problems [13,24]. Aligned with negative impressions of children by parents at risk for abuse [25], parents’ risk of child maltreatment is elevated when they attribute their child’s misbehavior to negative intent [26,27,28,29,30,31]. Indeed, parents’ negative intent attributions predict their later child abuse risk longitudinally [32], and mothers who have been identified as abusive report more negative child attributions than comparison parents [33]. Relative to a comparison group, mothers at risk to abuse were more inclined to view children’s behavior negatively, attribute negative child behaviors to internal causes (e.g., negative intent), and attribute positive behaviors to factors external (less dispositional) to the child [34]. Thus, parents at risk to abuse may not only be more inclined to view children negatively, they may also be less inclined toward positive impressions and attribute positive behaviors to more transitory child qualities [25]. Furthermore, mothers at risk to abuse appear more likely to become angry when ascribing negative intent to child misbehavior [21,35] and to respond coercively with children when experiencing anger [36,37], consistent with observed links between parental anger and child abuse risk noted across several studies [34,38,39].

However, limited attention has been given to how stress may influence such parental perceptions of their children, with even less work on parental coping. Some theories suggest that parents who engage in physical abuse may be more prone to view their children’s behavior as problematic, particularly when parents are stressed [36]. In a community sample, parents’ heightened stress was related to both their higher child abuse risk and their perception of more child behavior problems [14]. Negative child attributions also mediated the association between parental stress and harsh and abusive parenting in a community sample of parents [27]. However, an experimental study did not observe that induced situational stress evoked more negative child attributions in a community sample of parents [40]. Other early work linked parental stress with anger in relation to parents’ abuse risk [41,42], suggesting that reductions in negative affect from both stress and anger could decrease risk for abuse. A treatment program focused on both attributional retraining and anger management to reduce parents’ abuse risk demonstrated that parents perceived fewer child problem behaviors after treatment [43], consistent with other programs that incorporate parent training in regulating anger [44]. Such treatment effects suggest that reductions in parental anger and negative attributions can precipitate changes in parents’ perceptions of child behavior problems. Although training in anger regulation may be viewed as promoting parents’ coping, empirical work directly connecting parents’ coping with their perceptions of child behavior is lacking. Notable across studies is a heavy reliance on community and low-risk samples to draw inferences on how parents at high risk to engage in maltreatment may perceive their children.

Furthermore, current research relies on assessing potential risk or protective factors distal to actual parenting events. Thus, research relies on self-report questionnaires that prompt respondents to provide summative information about their experiences or beliefs—namely, their generalized impression across occasions. These approaches can thereby be subject to retrospective recall errors and gross overgeneralizations. Naturalistic assessments such as experience sampling methods (ESM) provide more intensive inquiry into processes occurring proximal to the behaviors of interest [45,46,47]. ESM can utilize written or electronic diary methods. Specifically, with regard to parental discipline, some early studies investigated reports about parental discipline episodes with written daily diaries, reviewing four weeks of questions [48] or qualitatively evaluating open-ended written diaries [49]. However, paper daily diaries sent home with parents require high literacy, can be burdensome, and are subject to recall biases if not completed at the designated time [50]. Electronic methods can reduce participant burden with quick administration, time-stamps to verify when questions were completed, and “branch” questions to allow skip patterns based on prior responses [46].

For example, one recent study conducted brief phone interviews with 55 mothers multiple times per day for six days, inquiring about mothers’ emotion regulation during parenting, including during challenging parent-child interactions; findings indicated that reports of emotions were tied more closely to their daily motivations than to their behaviors, with nuances for specific emotions that demonstrated the value of examining parental emotions in naturalistic environments [51]. Another Swiss study instructed parents to characterize their parenting behavior with their child as positive or negative using end-of-day assessments by reporting on hand-held devices [52], finding that mothers’ more stressful work days were associated with more negative parenting that day, and more positive parenting was reported by both mothers and fathers when they perceived greater spousal support. Yet another Swiss study involved 35 mothers completing the same self-report measure of parenting style daily for 10 days, indicating that such assessment was effective in characterizing changes in parenting across different discipline episodes [53]. An additional study administered end-of-day surveys for one week using a smartphone-based application to assess variability in harsh or positive parenting, indicating that parenting stress was associated with variability in harsh parenting [54]. Notably, the limited work on experience sampling thus far continues to focus on the perceptions of low-risk, community samples of mothers. 

Consequently, the present investigation conducted an intensive examination of mothers’ perceptions of daily stress and coping in relation to their perceptions of their children using experience sampling methods. A unique advantage of daily life methods is the ability to examine within-person variability. Moreover, another distinctive strength of daily life methods is the ability to estimate within-person relationships [46], which have a different and more incisive interpretation than the more commonly reported correlations involving between-person associations. For example, as a given mother’s stress increases, relative to their own average level of daily reported stress (i.e., when they are more stressed than usual), how does the outcome variable change? These within-person effects seek to explain within-person variability and are unconfounded by between-person individual differences (e.g., age, personality, or other factors that do not vary across the days; [55]). 

Furthermore, contrary to prior research that has relied on low-risk community samples of parents, the current study enrolled a high-risk sample of mothers who had been identified through child protective services for child maltreatment. These mothers completed daily diaries, reporting on their stress, coping, as well as their perceptions of their child’s behavior; mothers provided daily evaluations of how severe they judged child misbehavior, their anger in response to misbehavior, as well as their attributions regarding their children’s misbehavior or their children’s positive behavior. We explored the variability of maternal perceptions of their daily stress and coping as well as their perceptions of their children’s behavior. Based on prior research that has relied on summative self-reported data, we anticipated that mothers’ perceptions of greater severity of child misbehavior would be related to their reports of experiencing more anger and more negative child intent attributions. We also examined whether mothers who experienced more stress or lower coping in a given day were more likely to perceive child behavior as problematic, to report their child’s misbehavior was more severe, to ascribe more negative intent to their child’s misbehavior, and to report greater anger associated with the misbehavior; conversely, we investigated whether mothers who reported better coping or lower stress in a given day were more likely to characterize their child’s good behavior as attributable to the child’s good mood or personality.

## 2. Materials and Methods

### 2.1. Participants

The sample consisted of 23 mothers participating in the “Advancing Innovative Methods to Study Parenting” (AIMS-P) Study, conducted in Birmingham, Alabama, USA. The study recruited mothers through the county child protective services who had been indicated/substantiated for child maltreatment and consequently mandated to designated parent training programs for child welfare-involved parents (those indicated/substantiated for child neglect only typically receive alternative services). Thus, this sample is at-risk for maltreatment recurrence.

Mothers’ mean age was 29.70 years (*SD*_age_ = 4.88). Mothers self-identified their race: 82.6% Black, 17.4% White; 8.7% of this sample also identified as Latina and 4.3% also considered themselves biracial. Mothers’ reported their educational attainment: 34.8 less than high school, 26.1% high school degree, and 34.8% some college or technical school training. Median combined household income was reported as under $8000 annually, with most unemployed (73.9%) and most receiving public assistance (82.6%). Over 52% considered themselves single parents, with 56.5% reporting they were in a romantic relationship. Mothers had an average of 3.26 children but, for the purposes of this study, were asked to focus their reporting on the child they found most challenging; on average, the target child was 6.7 years old (*SD*_age_ = 4.09) with 82.6% identified as male.

### 2.2. Procedures

**Figure 1:**
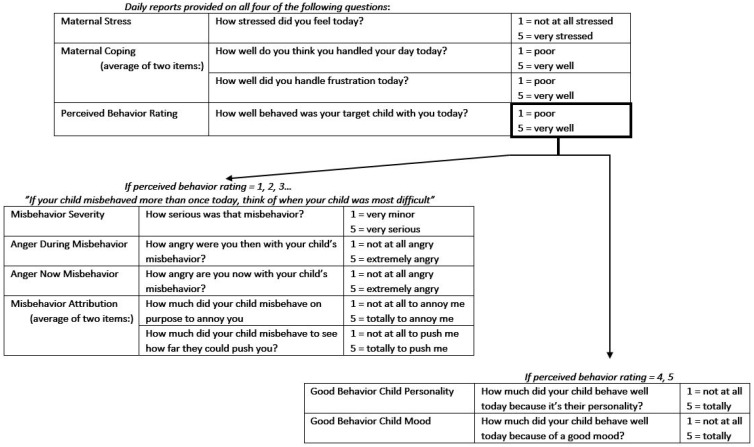
Items presented to mothers in a branching pattern.

ESM collection was administered through Metricwire, a data-collection service utilizing a smartphone-based app (www.metricwire.com, accessed on 29 October 2021). Metricwire signaled AIMS-P Study mothers to complete end-of-day daily diaries on their smartphones between 7–11 pm for 28 days, with a reminder signal within one hour if they had not completed the survey. AIMS-P participants received $30 for completing at least 21 end-of-day surveys. The median number of completed surveys was 24 (*M* = 22.52, *SD* = 7.06, range from 6 to 29). The ESM protocol for the AIM-P Study utilized a branching approach (see Figure 1 for a summary of how items in the current study utilized branching). Mothers first provided an assessment of their daily stress as well as their coping (the latter involved an average of two questions). Mothers were then asked whether they had spent at least one full waking hour with the target child that day. Mothers who reported spending at least one hour with their child were asked to characterize the target child’s behavior that day. Depending on their characterization of their child’s behavior, they either reported on child misbehavior (severity, anger during the misbehavior and currently, and an average score of two items on negative child intent attributions) or they reported on child good behavior (child personality or mood).

### 2.3. Analysis Plan

Daily diary data have a multilevel structure: the daily life items (the within-person, lower level) are nested within participants (the between-person, upper level). For data analysis, daily diary research typically uses multilevel models. Among their other virtues, multilevel models account for the nested data structure and accommodate different within-person sample sizes (i.e., some people will complete more surveys than others), which are two defining qualities of daily diary data [46,56]. The data files were restructured using R 4.1 [57], and the descriptive statistics, intraclass correlations, and within-person regression models were obtained using Mplus 8.1 [58]. 

Our primary analyses involve the within-person relationships between daily stress and coping (our day-level predictor variables) and outcomes related to their perceptions of child behavior. The correlations and regression coefficients are thus at the within-person level. 

## 3. Results 

### 3.1. Descriptive Statistics

Within-person descriptive statistics for the daily diary items are shown in Table 1. Due to conditional branching and occasional missing responses, the number of observations varies for different clusters of items (see Table 1). For the items about stress and coping, which were asked at the start of each survey, there were 518 observations. The mothers indicated that they had spent at least one waking hour with their child by the time of the survey around 94% of the time, which then yielded 485 observations for the item that asked for perceptions of the child’s behavior. The mothers usually rated their children as well behaved, so there were 101 observations for the items in the misbehavior branch (average number of responses per person of 5.61) and 383 observations for items in the good behavior branch (average number of responses of 16.65).

### 3.2. Correlations

Due to the multiple levels of branching and different item-level sample sizes, the within-person correlations between all the variables are not easily expressed in a single matrix. Ratings of stress and coping significantly correlated with each other (*r* = −0.47, *p* < 0.001), as one would expect, and perceived child behavior ratings significantly correlated with stress (*r* = −0.21, *p* = 0.005) and coping (*r* = 0.30, *p* < 0.001). Table 2 displays the within-person correlations for the child misbehavior items. As anticipated, perceptions that children’s misbehavior was more severe were significantly positively related to their anger and negative attributions; anger was also relatively persistent, with a significant relation between perceptions of experienced anger during the misbehavior episode as well as during the survey completion. The two child good behavior items correlated significantly with each other (*r* = 0.42, *p* < 0.001).

### 3.3. Within-Person Variability

Daily life methods permit an examination of within-person variability—in this case, how a mother’s perceptions, experiences, and attributions varied from day to day. Since the variables in Table 1 were repeatedly assessed, intraclass correlations can be used to describe how much of the variance is at the within-person level versus the between-person level [55]. Intraclass correlations vary from 0 to 1 and indicate the proportion of variance that is at the between-person level. Variables with higher ICCs are thus more “trait like” (the construct varies more person to person); variables with lower ICCs are more “state like” (the construct varies more day to day).

**Figure 2:**
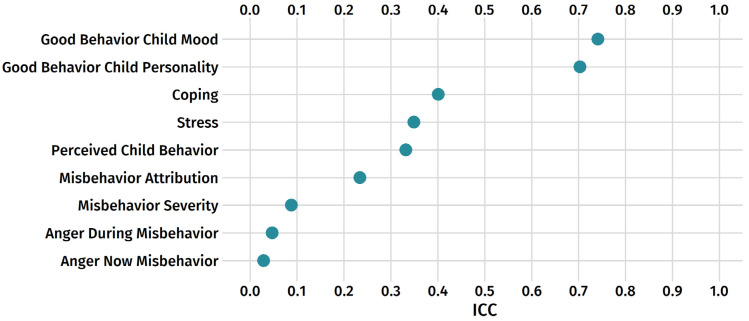
Intraclass correlations for daily reports (items sorted by largest to smallest).

Figure 2 displays the ICCs sorted from largest to smallest. The largest ICCs, both greater than 0.70, were for mothers’ attribution of positive behavior to the child’s personality or good mood. The high ICCs indicate that variation in these attributions was largely a matter of individual differences between mothers, not contextual factors that varied from day to day, implying mothers’ perceptions of more trait-like characteristics were ascribed for good behavior. Otherwise, the remaining ICCs were below 0.50, indicating that most of the variance was at the within-person, day-to-day level. The lowest ICCs were for ratings of anger and for the severity of misbehavior. Nearly all the variance was at the within-person level, which means that mothers’ ratings about themselves (stress and coping) and their children’s behavior (particularly misbehavior) were largely driven by factors that varied from day to day.

### 3.4. Within-Person Regression Models

We conducted a series of multilevel regression models to estimate the within-person relationships between maternal stress and coping (the two simultaneous predictor variables) and ratings of their children as outcome variables. The analyses were conducted using Mplus 8.1, using maximum likelihood estimation with robust standard errors. The predictors were group-mean centered—each mother’s scores were centered at their own mean, creating deviation scores—so the coefficients represent how an outcome is expected to change as a person changes from their own mean value [46,56]. Due to the relatively small number of mothers, the models were estimated as fixed rather than random. As an illustration, the effects of stress and coping on perceptions of the child’s behavior followed this specification [54]:

Level 1: *Y_ij_* = β_0*j*_ + β_1*j*_(Stress) + β_2*j*_(Coping) + *r_ij_*
Level 2: β_0*j*_ = γ_00_
  β_1*j*_ = γ_01_
  β_2*j*_ = γ_02_

Both raw regression coefficients and standardized coefficients are reported in Table 3.

When considered in concert with coping, maternal stress only significantly predicted their reports that they still felt angry about their child’s misbehavior that day (misbehavior severity only approached significance); mothers who reported better coping characterized their children’s behavior as more positive that day (child behavior rating) and were significantly more likely to ascribe their children’s positive behavior that day to their personality and good mood.

## 4. Discussion

The current investigation focused on the daily experiences of a sample of mothers who had been identified for child maltreatment and mandated to official parenting classes. Maternal stress and coping were interrelated and were related to perceptions of child behavior. Mothers’ ratings of their children’s good behavior were relatively “trait like” given their high ICCs, so these variables apparently vary more from mother to mother, much like a stable individual difference, rather than from day to day. However, their daily stress and coping as well as their perceptions regarding incidents of child misbehavior varied largely at the within-person level, so these variables appear more “state like” and sensitive to contextual and daily factors. Regression models indicated that whereas greater maternal stress was related to their reports of current anger, maternal coping was related to perceptions that their children had behaved well and that such good behavior was attributable to their child’s good mood and personality.

Prior research investigating at-risk parenting has relied on summative information regarding parents’ stress [13,14,15], coping [16,17,18], and perceptions of child behavior [13,14,20]. However, the value of experience sampling methods permits the consideration of how state-like versus trait-like such assessments may be. Our findings demonstrate substantial daily fluctuations observed regarding maternal stress, coping, and perceptions of child misbehavior; only perceptions regarding child good behavior with respect to personality and mood demonstrated stability—not the perceptions that represent a concern for most professionals seeking to avert child abuse. The positive attributions for children’s good behavior reflected both potentially transitory qualities (mood) and dispositional qualities (personality)—suggesting that mothers at risk to abuse may not distinguish these positive attributions as suggested in prior work with community samples [34]. This daily variability in stress, coping, and child misbehavior perceptions implies that these are potentially malleable, therapeutically modifiable dimensions. Continued research is needed to identify what adversities and protective qualities may contribute to such daily fluctuations to better support parents in such therapeutic interventions. An important implication is also apparent: extant data that gauge mothers’ stress and coping and their perceptions about their child’s misbehavior using summative, self-report measures would be unable to capture these state-like fluctuations, which may be most influential in maternal behavior during parent-child conflict.

The current findings also indicate that for perceptions of child misbehavior, maternal anger was relatively persistent during the course of the day (anger during the misbehavior was strongly correlated with current anger), suggesting that end-of-day reports of maternal anger can provide meaningful information of mothers’ negative affect during potential discipline scenarios earlier in the day. However, we did not assess the amount of time elapsed since the episode on which they were responding, which would be an important element to consider in future research to more clearly evaluate the persistence of negative affect. Such anger was also correlated with their perception that the misbehavior was severe and with more negative child intent attributions, consistent with prior research [21,35], with both anger [34,38,39] and negative child intent attributions [26,27,28,29,30,31] that have demonstrated robust links with greater abuse risk. 

Despite these links observed within perceptions of child misbehavior, our findings indicated that daily stress was not significantly related to negative child intent attributions, consistent with a recent experimental study wherein induced stress did not provoke negative child attributions [40]. When considered in concert with coping, maternal stress was only significantly related to reports that they remained angry, potentially reflecting mothers’ persistent negative affect state (both stress and anger) while completing the end-of-day survey. Mothers who reported handling their frustration and day well—our indicator of maternal coping—were also more likely to perceive their child was better behaved which they attributed to their child’s good mood and personality. Better emotion regulation and frustration tolerance have previously been associated with reduced likelihood for child abuse [15,19]. 

The temporal directions, however, in our analyses are unclear: whether mothers who believe their children behaved well that day in turn believe they coped well or whether mothers who believe they coped well now perceive their child behaved well that day, potentially ascribed to their child’s good mood and personality. What the findings do suggest, however, is that better coping covaries with judgments that their children are behaving appropriately which would decrease their likelihood of engaging in at-risk parenting behavior. If future longitudinal work can replicate these findings to clarify temporal directions, supporting mothers in developing better coping and regulatory strategies could facilitate their managing their daily stressors and challenging child behavior. Given the high sociodemographic risk of the current sample, mothers’ greater access to tangible and intangible resources and support systems may contribute to their perception of effective coping with the stressors they appear more likely to encounter. Furthermore, because the current analyses do not consider the directionality between parent and child behavior, future ESM research could expressly evaluate transactional processes whereby child behavior may evoke parental responses that in turn affect child behavior reactions (e.g., [59]), tracking changes within dyads across time.

### Limitations and Additional Future Directions

A number of limitations are worth noting, beginning with the limited sample size. Although this intensive investigation involved a welfare-involved sample of mothers, future studies should enroll larger sample sizes to provide greater insight into the experiences of parents at risk for abuse. Studies that signal parents more than once per day, without being intrusive or burdensome, may provide more precise insights into changes that may unfold during the course of the day, potentially providing reports even more proximal to parent-child interactions than end-of-day surveys. In particular, we did not assess the timing of the misbehavior episodes relative to when mothers were providing their daily reports, nor did we assess how much absolute time the mother had spent with the child; both timing components should be considered in future research to evaluate their effects and to consider the persistence of the constructs of interest (for example, whether anger or negative attributions are persistent if distal versus proximal to the reporting time). Mothers also only reported on attributions about child mood and personality for good behavior, not misbehavior; future work should consider whether these additional qualities are similarly stable for parents’ perceptions of misbehavior. Further, we did not inquire whether mothers had or had not experienced stress in a given day to utilize as a branching question to determine the extent to which coping mechanisms would be required—a possible nuance also worth considering in future work. In addition, mothers were not providing reports anonymously, which could conceivably reduce candor and induce social desirability responding, particularly as these mothers were already mandated to receive services; future work should consider mechanisms to provide parents the opportunity to provide reports anonymously.

Our sample of mothers was welfare-involved and thereby does not reflect the much wider population of parents who engage in child maltreatment who may never come to the attention of authorities. Furthermore, samples that include fathers would be particularly useful given the continued reliance in most research on maternal perceptions, despite the reality that a substantive proportion of maltreatment is perpetrated by fathers [2,3]. The current sample of mothers happened to select primarily male children as the target child they experienced as most difficult; although this gender imbalance is not reflected in official maltreatment statistics, it appears worth further investigation whether there are gender differences in parents’ perceptions of children’s behavior as challenging. A particularly interesting future direction would be to replicate these intensive daily diary methods examining parent-child dyads by gender to consider same-gender versus cross-gender differences in perceptions.

Continued research with at-risk samples is needed. Research with community samples operates on the assumption that such parents differ quantitatively, not qualitatively, from parents who engage in child maltreatment. However, if the effect sizes for different contributors to abuse risk are modest, some contributors may only become apparent with research examining high risk samples. Given the significant public health burden of child maltreatment, understanding how parents view their children can inform prevention and intervention programs which could reduce the likelihood of conflictual parent-child interactions.

## Figures and Tables

**Table 1:** Within-person descriptive statistics for maternal daily reports.

Variable	*M (SD)*	*Med*	*Min, Max*
Maternal Stress and Coping (*n* = 518)			
Maternal Stress	1.92 (1.22)	1	1, 5
Maternal Coping	4.03 (1.01)	4	1, 5
Child Behavior (*n* = 485)			
Perceived Child Behavior Rating	4.17 (0.99)	4	1, 5
Misbehavior (*n* = 101)			
Misbehavior Severity	2.20 (1.07)	2	1, 5
Anger During Misbehavior	2.40 (1.10)	2	1, 5
Anger Now Misbehavior	1.43 (0.68)	1	1, 3
Misbehavior Attribution	2.16 (1.14)	2	1, 5
Good Behavior (*n* = 383)			
Good Behavior Child Personality	4.25 (0.95)	5	1, 5
Good Behavior Child Mood	4.28 (0.98)	5	2, 5

**Table 2:** Within-person correlations for perceived child misbehavior.

	1.	2.	3.
1. Severity			
2. Anger During	0.63		
3. Anger Now	0.56	0.54	
4. Attribution	0.60	0.62	0.51

*Note.* All correlations are significant, *p* < 0.001.

**Table 3:** Summary of within-person regression of child ratings on maternal stress and coping.

	Maternal Stress			Maternal Coping		
	*b* (*SE*)	β	*p*	*b* (*SE*)	β	*p*
Perceived Child Behavior Rating	−0.08 (0.05)	−0.09	0.127	0.27 (0.07)	0.25	<0.001
Misbehavior Severity	0.13 (0.08)	0.12	0.091	−0.11 (0.07)	−0.09	0.120
Anger During Misbehavior	0.15 (0.15)	0.13	0.331	−0.10 (0.12)	−0.09	0.368
Anger Now Misbehavior	0.13 (0.07)	0.19	0.050	0.00 (0.06)	0.00	0.992
Misbehavior Attribution	0.13(0.14)	0.13	0.342	−0.04 (0.08)	−0.04	0.601
Good Behavior Child Personality	0.03 (0.03)	0.05	0.464	0.09 (0.04)	0.12	0.010
Good Behavior Child Mood	0.04 (0.03)	0.08	0.190	0.13 (0.04)	0.20	<0.001

*Note.* Column “*b* (*SE*)” reports the unstandardized regression weight and its standard error; “β” reports the standardized regression weight; and “*p*” reports the *p*-value of the standardized regression weight. Predictors were group-mean centered (i.e., centered at each participant’s own mean).

## Data Availability

The data files and input files are publicly available at Open Science Framework: https://osf.io/n54py/, accessed on 30 October 2021.
